# Characteristics analysis of 157 cases of central airway stenosis due to tracheobronchial tuberculosis: A descriptive study

**DOI:** 10.3389/fpubh.2023.1115177

**Published:** 2023-02-02

**Authors:** Rongjuan Zhuang, Mingjin Yang, Li Xu, Yishi Li, Ying Li, Tingting Hu, Yan Chen, Xiao Nie, Xiaofeng Yan, Xianghua Kong, Song Yang, Shuliang Guo

**Affiliations:** ^1^Department of Respiratory and Critical Care Medicine, The First Affiliated Hospital of Chongqing Medical University, Chongqing, China; ^2^Department of Respiratory and Critical Care Medicine, The First Affiliated Hospital of Chongqing Medical and Pharmaceutical College, Chongqing, China; ^3^Department of Respiratory and Critical Care Medicine, Affiliated Hospital of North Sichuan Medical College, Nanchong, China; ^4^Department of Tuberculosis, Chong Qing Public Health Medical Center, Chongqing, China; ^5^Department of Comprehensive Internal Medicine, Chong Qing Public Health Medical Center, Chongqing, China

**Keywords:** tracheobronchial tuberculosis, central airway stenosis, bronchoscopic findings, tracheobronchomalacia, clinical characteristics

## Abstract

**Background:**

Tracheobronchial stenosis, particularly central airway stenosis, which frequently results in severe complications such as lung damage, occurs in patients with tracheobronchial tuberculosis (TBTB).

**Objectives:**

To analyze the clinical characteristics of patients with central airway stenosis due to tuberculosis (CASTB).

**Methods:**

Retrospective analysis was performed on the clinical features, radiological features, bronchoscopic features and treatment of 157 patients who were diagnosed with CASTB in two tertiary hospitals in Chongqing, China, from May 2020 to May 2022.

**Results:**

CASTB mostly occurs in young patients and females. Patients with CASTB exhibited different symptoms repeatedly during the disease, especially varying degrees of dyspnea, prompting many patients to undergo bronchoscopic intervention and even surgery. Patients with cicatricial strictures constituted the highest proportion of the TBTB subtype with CASTB and 35.7% of the patients with CASTB were found to have tracheobronchomalacia (TBM) under bronchoscopy. CASTB and TBM mainly involved the left main bronchus. Patients with lower levels of education had higher rates of TBM. Patients with TBM manifested shortness of breath more frequently than patients without TBM. Patients with TBTB who had undergone bronchoscopic interventions have a higher rate of TBM.

**Conclusions:**

Despite mostly adequate anti-tuberculosis chemotherapy, patients with TBTB can present with CASTB involving severe scarring stenosis, bronchial occlusion, tracheobronchomalacia and even destroyed lung.

## 1. Introduction

The global tuberculosis report 2022 points out that tuberculosis (TB) is still ranked in the top 10 causes of death worldwide. According to the latest World Health Organization statistics, there are approximately 10.6 million people with TB disease, and 1.6 million deaths were due to TB in 2021 (1).

Tracheobronchial tuberculosis (TBTB) is a special type of TB infecting the tracheobronchial tree that could affect any portion or layer of the tracheobronchial wall (2). Published evidence suggests that TBTB may be present in as many as 40% of patients with pulmonary tuberculosis (PTB) (3–5). The incidence of airway stenosis associated with TBTB can be as high as 68% during the first 4–6 months of illness (6). However, due to the lack of specific symptoms and the lack of attention from patients and medical staff, some cases of early reversible airway stenosis may progress gradually and eventually result in permanent cicatricial stenosis. If the stenosis occurs in the central airway, it is called central airway stenosis due to tuberculosis (CASTB), which is the most severe type because it can lead to varying degrees of respiratory distress, recurrent respiratory infections, and even death from asphyxia (7–9).

However, the clinical characteristics of CASTB are relatively rarely reported in the literature. Consequently, we aimed to retrospectively analyze the clinical characteristics of patients with CASTB to increase physician awareness of the clinical features and harmfulness of the condition.

## 2. Materials and methods

### 2.1. Law and ethics

This study is a retrospective case study approved by the Medical Ethics Committee of the First Affiliated Hospital of Chongqing Medical University (No. 20188501) without the need for participants' explicit consent. The institutional review board of each hospital approved the analysis of patients' clinical, radiological, and bronchoscopic data.

### 2.2. Study design

We performed a retrospective analysis of patients with CASTB treated at the First Affiliated Hospital of Chongqing and the Public Health Medical Center (PHMC) from May 2020 to May 2022. The inclusion criteria were as follows: (1) patients diagnosed with TBTB based on positive acid-fast bacilli smear or culture of *Mycobacterium tuberculosis* (Mtb) or histopathologically and with endoscopic findings suggestive of TBTB (9); (2) patients with proven TBTB with endoscopic findings of central airway stenosis (including trachea, right and left main bronchi and right middle segment bronchi) (10, 11). The exclusion criteria were as follows: (1) patients with tracheobronchial stenosis secondary to etiologies other than TB; (2) patients with incomplete clinical data; (3) patients diagnosed with life-threatening cardiovascular, hepatic, hematopoietic, or other serious diseases.

According to the Chinese guidelines for the classification of TBTB (9), bronchoscopic subtypes of TBTB may be classified as inflammatory infiltration, ulceration necrosis, granulation hyperplasia, cicatricial stricture, tracheobronchomalacia and lymphatic fistula. Endobronchial stenosis was evaluated by comparing the decrease in cross-sectional area and grade under bronchoscopy as follows: Grade I (<50%); Grade II (51–70%); Grade III (>70%) (12). Clinical case notes and electronic medical records of all patients were reviewed. Demographic information, symptoms, radiological features, bronchoscopic findings, and treatment were recorded.

### 2.3. Statistical analysis

Statistical analyses were performed using SPSS version 26.0 (SPSS Inc, Chicago, IL, USA), with *P* < 0.05 being statistically significant (all *P*-values are from two-sided tests). Normality was tested using the Shapiro-Wilk test separate parametric and non-parametric variables. The continuous variables of normally distributed data are expressed as means + standard deviation and differences between any two groups were assessed by an unpaired *t*-test. Categorical data are expressed as numbers (percentage), and comparisons for testing statistically significant differences were made using the χ^2^-test (minimum expected values ≥5) or Fisher's exact test (minimum expected values <5).

## 3. Results

### 3.1. Characteristics of patients with CASTB

The study included 157 patients. Of the patients diagnosed with CASTB (mean age 32.5 years, range 17–64 years), 132 were female (84.1%, median age 32.4 years) and 25 were male (15.9%, mean age 33.4 years). The proportion of patients aged 20–29 years diagnosed with CASTB was significantly higher than that of other age groups, followed by the 30–39 age group of patients. Thirty-three patients diagnosed with CASTB (21.0%) were underweight (BMI < 18.5 kg/m^2^), 14 (8.9%) were the national minority, 36 (22.9%) lived in rural areas, 99 (63.1%) had high school or lower education level, 104 (66.2%) were married, 7 (4.5%) had smoking history, 3 (1.9%) had diabetes, 40 (25.5%) had a history of PTB, 14 (8.9%) had drug resistance and 18 (11.5%) had irregular anti-TB treatment. The common symptoms of CASTB included cough (132/157, 84.1%), expectoration (118/157, 75.2%), shortness of breath (91/157, 58.0%), hemoptysis (29/157, 18.5%) and weight loss (40/157, 25.5%). One hundred and twenty-three patients (78.3%) underwent bronchoscopic intervention (including cryotherapy, electric coagulation, balloon dilatation, and stent insertion) and 2 of them underwent surgery. The remaining 34 patients were conservatively managed (Table 1).

**Table 1:** Demographic and clinical characteristics of 157 patients diagnosed with CASTB.

	**Patients (*n* = 157)**
Age [mean (SD)]	32.5 (11.41)
**Age categories (y)**	
<20	10 (6.4%)
20–29	73 (46.5%)
30–39	32 (20.4%)
40–49	24 (15.3%)
50–59	15 (9.6%)
≥60	3 (1.9%)
BMI [mean (SD)]	20.93 (3.12)
**BMI (kg/m** ^2^ **)**	
<18.5 (underweight)	33 (21.0%)
18.5–24.9 (normal weight)	113 (72.0%)
25.0–29.9 (overweight)	9 (5.7%)
≥30.0 (obese)	2 (1.3%)
**Gender**	
Male	25 (15.9%)
Female	132 (84.1%)
**National minority**	
Yes	14 (8.9%)
No	143 (91.9%)
**Residence**	
Urban	121 (77.1%)
Rural	36 (22.9%)
**Education**	
College or above	58 (36.9%)
High school or less	99 (63.1%)
**Marital status**	
Married	104 (66.2%)
Spinsterhood	53 (33.8%)
**Smoking history**	
Never-smoker	150 (95.5%)
Former or current smoker	7 (4.5%)
**Diabetes**	
Yes	3 (1.9%)
No	154 (98.1%)
**History of PTB**	
Yes	40 (25.5%)
No	117 (74.5%)
**Drug resistance**	
Yes	14 (8.9%)
No	85 (54.1%)
Yes	18 (11.5%)
No	139 (88.5%)
**Symptoms at presentation**	
**Cough**	
Yes	132 (84.1%)
No	25 (15.9%)
**Expectoration**	
Yes	118 (75.2%)
No	39 (24.8%)
**Shortness of breath**	
Yes	91 (58.0%)
No	66 (42.0%)
**Hemoptysis**	
Yes	29 (18.5%)
No	128 (81.5%)
**Weight loss**	
Yes	40 (25.5%)
No	117 (74.5%)
**Treatment methods**	
Bronchoscopic intervention	123 (78.3%)
Cryotherapy	120 (76.4%)
Electric coagulation	94 (59.9%)
Balloon dilatation	104 (66.2%)
Stent insertion	13 (8.3%)
Surgical operation	2 (1.3%)

Data are expressed as numbers (percentages). CASTB, central airway stenosis due to tracheobronchial tuberculosis; BMI, body mass index.

### 3.2. Chest CT and bronchoscopy

The results of chest CT and bronchoscopy are presented in Table 2. Based on chest CT, 51 (32.5%) patients showed tracheobronchial wall thickening, 136 (86.6%) showed tracheobronchial stenosis, 33 (21.0%) patients showed mediastinal nodes increase, 34 (21.7%) patients showed mediastinal nodes calcification and 19 (12.1%) patients showed cavities. Of all the study patients, 5 (3.2%) had destroyed lung and two of them underwent a surgical operation. The results of bronchoscopy indicated 101 (64.3%) cases of cicatricial strictures, 7 (4.5%) cases of TBM, and 49 (31.2%) mixed cicatricial strictures and TBM. The lesions were mainly located in the left main bronchus (58.0%), followed by the right bronchus (30.6%), the left upper lobe bronchus (26.8%), and the right upper lobe bronchus (23.6%). Patients with multiple lesions under bronchoscope constituted 77.1%. A total of 56 (35.7%) of the 157 patients with CASTB manifested TBM and the highest proportion of TBM lesions was the left main bronchus (37/56, 66.1%). Of these patients including all lesions detected bronchoscopically, 20 (12.7%) were mild stenosis, 56 (35.7%) were intermediate stenosis, and 81 (51.6%) were severe stenosis.

**Table 2:** Chest CT and bronchoscopy features of 157 patients with CASTB.

	**Patients (*n* = 157)**
**Chest CT features**	
Tracheobronchial wall thickening	51 (32.5%)
Tracheobronchial stenosis	136 (86.6%)
Mediastinal nodes increase	33 (21.0%)
Mediastinal nodes calcification	34 (21.7%)
Cavity	19 (12.1%)
Destroyed lung	5 (3.2%)
**Bronchoscopic features**	
**Type of TBTB**	
Cicatricial stricture	101 (64.3%)
Tracheobronchomalacia	7 (4.5%)
Cicatricial stricture + Tracheobronchomalacia	49 (31.2%)
**Site of lesions**	
Trachea	29 (18.5%)
LMB	91 (58.0%)
RMB	48 (30.6%)
RBI	33 (21.0%)
LULB	42 (26.8%)
LLLB	23 (14.6%)
RULB	37 (23.6%)
RMLB	15 (9.6%)
RLLLB	15 (9.6%)
**Number of lesions involved**	
Single	36 (22.9%)
Multiple	121 (77.1%)
**Site of TBM lesions**	
Trachea	5 (3.2%)
LMB	37 (23.6%)
RMB	10 (6.4%)
RMI	8 (5.1%)
**Grade of tracheobronchial**	
**Stenosis (including all bronchi)**	
Grade 1	20 (12.7%)
Grade 2	56 (35.7%)
Grade 3	81 (51.6%)

Data are expressed as numbers (percentages). CASTB, central airway stenosis due to tracheobronchial tuberculosis; CT, computed tomography; LMB, left main bronchus; RMB, right main bronchus; RBI, right bronchus intermedius; LULB, left upper lobe bronchus; LLLB, left lower lobe bronchus; RULB, right upper lobe bronchus; RMLB, right middle lobe bronchus; RLLB, right upper lobe bronchus; TBM, tracheobronchomalacia.

### 3.3. Comparison of TBM group vs. non-TBM group

Patients with lower levels of education exhibited a higher incidence of TBM compared with patients with higher levels of education (*p* = 0.008; Table 3). A greater percentage of patients with TBM experienced shortness of breath (73.2 vs. 49.5%, *p* = 0.004) than patients without TBM. Not only that, a greater percentage of patients with TBM had modified Medical Research Council (mMRC) dyspnea scores ≥ 2 (30.4 vs. 12.9%, *p* = 0.008) than patients without TBM. A larger proportion of patients with TBM had ever undergone bronchoscopic interventions (92.3 vs. 70.3%, *p* = 0.001) than patients without TBM. No statistically significant difference was found in the number of lesions and degree of stenosis under bronchoscope between the two groups (*P* > 0.05; Table 3).

**Table 3:** Comparison of TBM group (*n* = 56) vs. non-TBM group (*n* = 101).

	**TBM** **(*n* = 56)**	**Non-TBM** **(*n* = 101)**	***P*** **-value**
Age[mean (SD)]	32.52 (1.56)	32.63 (1.13)	0.952
BMI [mean (SD)]	21.18 (0.43)	20.79 (0.31)	0.456
**Gender**			0.160
Male	12 (21.4%)	1,312.9 (%)	
Female	44 (78.6%)	88 (87.1%)	
**Residence**			0.950
Urban	43 (76.8%)	78 (77.2%)	
Rural	13 (23.2%)	23 (22.8%)	
**Education**			0.008
College or above	13 (23.2%)	45 (44.6%)	
High school or less	43 (76.8%)	56 (55.4%)	
**Symptoms**			
**Cough**			0.970
Yes	47 (83.9%)	85 (84.2%)	
No	9 (16.1%)	16 (15.8%)	0.973
**Expectoration**			
Yes	42 (75.0%)	76 (75.2%)	
No	14 (25.0%)	25 (24.8%)	
**Hemoptysis**			0.254
Yes	13 (23.2%)	16 (15.8%)	
No	43 (76.8%)	85 (84.2%)	
**Shortness of breath**			
Yes	41 (73.2%)	50 (49.5%)	0.004
No	15 (26.8%)	51 (50.5%)	
**Weight loss**			0.296
Yes	17 (30.4%)	23 (22.8%)	
No	39 (69.6%)	78 (77.2%)	
**mMRC dyspnea scores** ≥**2**			0.008
Yes	17 (30.4%)	13 (12.9%)	
No	39 (69.6%)	88 (87.1%)	
**Treatment methods**			
Bronchoscopic intervention	52 (92.3%)	71 (70.3%)	0.001
Cryotherapy	51 (91.1%)	69 (68.3%)	0.001
Electric coagulation	39 (69.6%)	55 (54.5%)	0.063
Balloon dilatation	45 (80.4%)	59 (58.4%)	0.005
Stent insertion	5 (8.9%)	8 (7.9%)	0.826
Surgical operation	1 (1.8%)	1 (1.0%)	0.670
**Number of lesions involved under bronchoscope**			0.211
Single	16 (28.6%)	20 (19.8%)	
Multiple	40 (71.4%)	81 (80.2%)	
Grade 1	3 (5.4%)	17 (16.8%)	
Grade 2	22 (39.3%)	34 (33.7%)	
Grade 3	31 (55.4%)	50 (49.5%)	

Continuous data are expressed as means ± standard. Differences between the 2 groups were tested by the unpaired t-test for normally distributed continuous variables. Categorical data are expressed as numbers (percentage), and comparisons for testing statistically significant differences were made using the χ^2^ test (minimum expected values ≥5) or Fisher's exact test (minimum expected values <5). TBM, tracheobronchomalacia; BMI, body mass index; mMRC, Medical Research Council; LMB, left main bronchus; RMB, right main bronchus; RBI, right bronchus intermedius.

## 4. Discussion

This study was the first dual-center, retrospective report analyzing the clinical characteristics of CASTB. This study found that CASTB mostly occurs in young patients and females. Patients with CASTB manifested different symptoms repeatedly during the disease, especially varying degrees of dyspnea, prompting many patients to undergo bronchoscopic intervention and even surgery. The TBTB subtypes of patients with CASTB which accounted for the highest proportion were cicatricial strictures and 35.7% of the patients with CASTB were found to have TBM under bronchoscope. The left main bronchus was more prone to be involved in patients with CASTB and TBM. Patients with lower levels of education had higher rates of TBM. Patients with TBM experienced shortness of breath more frequently than patients without TBM. Patients with TBTB who had undergone bronchoscopic interventions had a higher rate of TBM. Of the 157 patients with CASTB, 68.8% had cicatricial stricture, 35.7% had tracheobronchomalacia and 3.2% had destroyed lung.

CASTB is seldom reported. In 2018, a large-scale prospective study (13) enrolled 392 patients with scarring airway stenosis from 18 tertiary hospitals. It reported that TBTB was the most common cause of scarring airway stenosis in Chinese adults with a high rate of incidence in young women. Similar sex- and age-related preponderance was observed in our study of CASTB. In the present study, nearly half of the patients were aged between 20 and 29 years probably due to the stronger immunity and inflammatory response to the disease, which may increase the risk of airway injury, even leading to permanent scar stenosis. The study found a higher proportion of females in patients with CASTB, which was consistent with previous reports (4, 14, 15). The bronchi were smaller and thinner in adult women than in men, and sputum retention promoted chronic infection of the bronchial lumen with Mtb (15). In addition, Gan et al. (16) suggested that estradiol may play an important role in the pathogenesis of TBTB by binding to estrogen receptor α (ERα) and affecting Mtb proliferation in bronchial epithelial cells. Estrogen is known to increase the levels of transforming growth factor β1 (TGF-β1) and fibronectin, which affects the wound healing response (17, 18). This may also explain the occurrence of severe cicatricial strictures in TBTB mostly in younger women.

In 2018, a large prospective study in southern China found that the proportion of retreated patients was 13.2% of the 1,442 patients with PTB, while this proportion was 10.7% among patients with TBTB (4). However, our study indicated that 25.5% of patients with CASTB had a history of PTB probably due to the significantly reduced cellular immune function in patients with retreated PTB than in newly treated patients (19).

During the disease, 21% of patients with CASTB were underweight and 25.5% of patients manifested symptoms of weight loss. TB itself is a wasting disease. However, changes in weight which may reflect disease activity, are not often the focus of outpatient consultations. Patients with CASTB manifested different symptoms during the disease, especially varying degrees of dyspnea, which was the main reason why patients underwent bronchoscopic interventions and even surgery. Most patients' symptoms were relieved after active treatment. However, patients with severe scarring stenosis, bronchial occlusion, malacia, and even lung damage, were still detected.

Our findings showed that the highest proportion of TBTB subtypes was cicatricial strictures (64.3%). The proportion of patients who had multiple lesions under bronchoscopy was as high as 77.1%, suggesting that the study patients had severe and extensive tracheobronchial lesions during the disease. The higher frequency of left bronchial involvement with CASTB and TBM is also consistent with previous studies (4, 13, 20). This finding may be explained by the location of the left main bronchus anatomically adjacent to the aortic arch and vulnerability to compression, increasing the risk of endobronchial infection of the left main bronchus (2).

This study found that 35.7% of the patients with CASTB had TBM and patients with lower levels of education had higher rates of TBM, which might be related to the fact that patients with lower levels of education had poorer compliance with standardized anti-tuberculosis treatments. Compared with patients without TBM, those with TBM were more likely to experience dyspnea because of the weakening of the airway wall and dynamic collapse of the airway lumen during respiration (21). Our analysis also found that patients with previous bronchoscopic interventions had a higher rate of TBM. There are two possibilities to be considered. One explanation is that this is an accidental phenomenon. Another possible explanation is that bronchoscopic intervention as a type of surgical procedure may damage cartilage or normal structures further contributing to the development of TBM. The impact of bronchoscopic interventions in the longer term is less predictable although it is a logical approach to relieve symptoms. Additional studies are needed to investigate the outcomes of bronchoscopic interventions, both immediate and in the longer term.

Some limitations of this study should be noted. First, this study was a retrospective study with a relatively small sample size, suggesting the possibility of bias. Second, lesions were graded based on the results of bronchoscopic examinations. It was possible that lesions were over or underestimated due to the subjective judgment of the physicians performing bronchoscopy despite similar evaluation criteria. Third, this study could not identify the predictors of TBM in patients with CASTB definitively. Further prospective, multicenter, and large-scale studies are needed to establish the incidence and predictors of CASTB and TBM in patients with TBTB.

## 5. Conclusion

Despite mostly adequate anti-tuberculosis chemotherapy, patients with TBTB can present with CASTB involving severe scarring stenosis, bronchial occlusion, tracheobronchomalacia, and even destroyed lung. Future studies should focus on the pathogenesis of bronchial fibrosis and bronchomalacia to prevent tracheobronchial stenosis at an early stage.

## Data availability statement

The datasets presented in this article are not readily available because the raw data supporting the conclusions of this article will be made available by the authors, without undue reservation. Requests to access the datasets should be directed to RZ, raphaela720@163.com.

## Ethics statement

The studies involving human participants were reviewed and approved by the Medical Ethics Committee of The First Affiliated Hospital of Chongqing Medical University (No. 20188501). Written informed consent from the participants' legal guardian/next of kin was not required to participate in this study in accordance with the national legislation and the institutional requirements.

## Author contributions

The data collection of the project was done by RZ, YinL, YC, TH, and XN. The idea for the paper, the data cleaning, the data analysis, and the writing was done by RZ and MY. The review of the paper and suggested ideas were done by LX, YisL, XY, XK, and SY. The review and final edits of the paper were done by RZ and SG. All authors contributed to the article and approved the submitted version.
