# Supplementary material for: Characteristics analysis of 157 cases of central airway stenosis due to tracheobronchial tuberculosis: A descriptive study
Source: Front Public Health. 2023 Feb 2;11:1115177. doi: 10.3389/fpubh.2023.1115177 (PMC9932966; doi:10.3389/fpubh.2023.1115177)
Supplement: Supplementary file 1 [file Table_1.docx]

Table S1. Bronchoscopic site of lesions of 157 patients with CASTB.

| **Site of lesions** | **Patients (n=157)** |
| --- | --- |
| LMB | 28(17.8%) |
| RMB | 1(0.6%) |
| RBI | 7(4.5%) |
| Trachea+LMB | 2(1.3%) |
| Trachea+RMB | 3(1.9%) |
| Trachea+RBI | 2(1.3%) |
| LMB+RMB | 1(0.6%) |
| LMB+LULB | 30(19.1%) |
| LMB+LLLB | 12(7.6%) |
| LMB+RULB | 1(0.6%) |
| RMB+RBI | 2(1.3%) |
| RMB+RULB | 15(9.6%) |
| RBI+RULB | 1(0.6%) |
| RBI+RMLB | 4(2.5%) |
| RBI+RLLB | 5(3.2%) |
| Trachea+LMB+RMB | 1(0.6%) |
| Trachea+LMB+LULB | 2(1.3%) |
| Trachea+LMB+LLLB | 3(1.9%) |
| Trachea+RMB+RULB | 8(5.1%) |
| Trachea+RMB+RLLB | 1(0.6%) |
| Trachea+RBI+RULB | 1(0.6%) |
| LMB+RMB+RULB | 1(0.6%) |
| LMB+LULB+LLLB | 6(3.8%) |
| RMB+RBI+RULB | 2(1.3%) |
| RMB+RBI+RMLB | 3(1.9%) |
| RMB+RBI+RLLB | 2(1.3%) |
| RMB+LULB+RULB | 1(0.6%) |
| RMB+RMLB+RLLB | 1(0.6%) |
| RBI+RMLB+RLLB | 1(0.6%) |
| Trachea+LMB+RMB+RULB | 2(1.3%) |
| Trachea+LMB+LULB+LLLB | 2(1.3%) |
| RMB+RBI+RMLB+RLLB | 1(0.6%) |
| RMB+RULB+RMLB+RLLB | 1(0.6%) |
| RBI+RULB+RMLB+RLLB | 1(0.6%) |
| Trachea+RMB+RULB+RMLB+RLLB | 2(1.3%) |
| RBI+LULB+RULB+RMLB+RLLB | 1(0.6%) |

Data are expressed as numbers (percentages). LMB-left main bronchus; RMB-right main bronchus; RBI-right bronchus intermedius; LULB-left upper lobe bronchus; LLLB--left lower lobe bronchus; RULB-right upper lobe bronchus; RMLB-right middle lobe bronchus; RLLB-right upper lobe bronchus.
